# Flesh Shear Force, Cooking Loss, Muscle Antioxidant Status and Relative Expression of Signaling Molecules (Nrf2, Keap1, TOR, and CK2) and Their Target Genes in Young Grass Carp (*Ctenopharyngodon idella*) Muscle Fed with Graded Levels of Choline

**DOI:** 10.1371/journal.pone.0142915

**Published:** 2015-11-23

**Authors:** Hua-Fu Zhao, Lin Feng, Wei-Dan Jiang, Yang Liu, Jun Jiang, Pei Wu, Juan Zhao, Sheng-Yao Kuang, Ling Tang, Wu-Neng Tang, Yong-An Zhang, Xiao-Qiu Zhou

**Affiliations:** 1 Animal Nutrition Institute, Sichuan Agricultural University, Chengdu 611130, China; 2 Fish Nutrition and Safety Production University Key Laboratory of Sichuan Province, Sichuan Agricultural University, Chengdu 611130, China; 3 Key Laboratory for Animal Disease-Resistance Nutrition of China Ministry of Education, Sichuan Agricultural University, Chengdu 611130, China; 4 Animal Nutrition Institute, Sichuan Academy of Animal Science, Chengdu 610066, China; 5 Institute of Hydrobiology, Chinese Academy of Sciences, Wuhan 430072, China; Temple University, UNITED STATES

## Abstract

Six groups of grass carp (average weight 266.9 ± 0.6 g) were fed diets containing 197, 385, 770, 1082, 1436 and 1795 mg choline/kg, for 8 weeks. Fish growth, and muscle nutrient (protein, fat and amino acid) content of young grass carp were significantly improved by appropriate dietary choline. Furthermore, muscle hydroxyproline concentration, lactate content and shear force were improved by optimum dietary choline supplementation. However, the muscle pH value, cooking loss and cathepsins activities showed an opposite trend. Additionally, optimum dietary choline supplementation attenuated muscle oxidative damage in grass carp. The muscle antioxidant enzyme (catalase and glutathione reductase did not change) activities and glutathione content were enhanced by optimum dietary choline supplementation. Muscle cooking loss was negatively correlated with antioxidant enzyme activities and glutathione content. At the gene level, these antioxidant enzymes, as well as the targets of rapamycin, casein kinase 2 and NF-E2-related factor 2 transcripts in fish muscle were always up-regulated by suitable choline. However, suitable choline significantly decreased Kelch-like ECH-associated protein 1 a (Keap1a) and Kelch-like ECH-associated protein 1 b (Keap1b) mRNA levels in muscle. In conclusion, suitable dietary choline enhanced fish flesh quality, and the decreased cooking loss was due to the elevated antioxidant status that may be regulated by Nrf2 signaling.

## Introduction

Fish meat, which could provide balanced amino acids, is an important protein source for humans [1]. Moreover, fish meat is rich in polyunsaturated fatty acids and vitamins, which is well accepted by consumers [2]. Currently, an inconvenient factor for consumer acceptance is fish flesh quality. Flesh quality deterioration may cause problems for the industry [3], which leads to tremendous economic losses for producers and poor consumption [4]. Cooking loss and firmness are technologically important characteristics of flesh quality [5]. Minimal weight loss after cooking and a firmness of flesh are desired by consumers, soft flesh leads to reduced acceptability [6]. Therefore, we have chosen cooking loss and firmness as focus points. Several studies demonstrated that fish flesh quality could be improved by dietary nutrients [7]. One study reported that vitamin E supplementation was shown to be effective at improving the flesh quality of rainbow trout [8]. Choline was discovered to be an essential vitamin for fish, which acts as an important methyl donor and component of acetylcholine [9]. Studies have shown that feeding a choline-deficient diet induced growth retardation and poor feed efficiency in juvenile Jian carp (*Cyprinus carpio* var. Jian) [10]. Fish growth primarily depends on the growth of muscle [11]. Craig and Gatlin [12] reported that dietary choline elevated muscle growth and muscle lipid content in juvenile red drum (*Sciaenops ocellatus*). Meanwhile, Mai et al. [13] showed that dietary choline increased muscle lipid content in juvenile cobia. However, there is no study on how dietary choline affects flesh quality in animal. Choline was recognized as a component of phospholipids that maintain cell membrane integrity [14]. Asghar et al. [15] reported that reduced phospholipid content increased the drip loss in pork muscle. Meanwhile, betaine is an important metabolite of choline [14]. Matthews et al. [16] showed that dietary betaine supplementation increased pH and decreased cooking loss and the firmness of pig muscle. A previous study found that dietary choline deficiency could significantly decrease vitamin E content in rat liver [17]. Moreover, the muscle choline concentration of juvenile cobia was significantly increased with dietary choline [13]. These observations indicated that dietary choline might have a positive effect on flesh quality, which warrants further investigation.

Firmness is an important flesh quality trait that influences acceptance of meat purchases in fish [18]. It was implied that the flesh firmness is associated with the collagen content in fish [19,20]. Johnston et al. showed that high collagen content contributed to muscle firmness in Atlantic salmon [21]. Meanwhile, Martinez et al. [22] reported that fish flesh firmness was negatively correlated with cathepsin activity in Atlantic salmon. However, less attention has been paid to the effects of dietary choline on flesh firmness through regulating muscle collagen content and cathepsin activity in animals. In rats, choline was found to increase liver vitamin C content [17], which improved collagen synthesis in cultured vascular smooth muscle cells [23], thereby improving muscle firmness. Moreover, in mice, choline significantly reduced lung interleukin-4 (IL-4) secretion [24], which down-regulated cathepsin B activity in mice macrophages [25]. The above studies indicated that choline may improve flesh firmness by increasing collagen content and decreasing cathepsin activity in fish muscle, which is worthy of more investigation.

Muscle pH is another important flesh quality parameter, and the development of flesh quality is positively correlated with post-mortem pH in fish muscle [26]. However, no studies have focused on the effects of choline on the pH of fish muscle. Betaine, a metabolic product of choline, increased the serum lactic acid content in newborn piglets [27]. Based on these studies, we speculate that dietary choline may influence the fish flesh pH, which warrants further investigation.

Water-holding capacity (WHC) is another key flesh quality characteristic that can affect the yield and quality of processed meat [28]. Several studies have demonstrated that meat WHC is associated with muscle oxidation damage. Oxidation damage significantly increased a loss of WHC in beef muscle [29]. Previous study in our laboratory has demonstrated that optimum zinc supplementation improved grass carp muscle WHC through attenuating muscle oxidative damage [30]. However, no research has focused on the effects of choline on flesh WHC, and whether dietary choline can affect WHC by affecting the oxidation damage in animal muscle is unknown. A study has shown that dietary choline deprivation could increase oxidative damage of rat kidney [31]. These findings indicated that choline might improve flesh WHC through decreasing muscle oxidation damage in fish. In addition, the oxidative damage of fish was induced by reactive oxygen species (ROS) [32]. To scavenge ROS, fish have developed antioxidant systems that, in general, are composed of the non-enzymatic compound glutathione (GSH) and antioxidant enzymes such as superoxide dismutase (SOD), glutathione peroxidase (GPx) and glutathione S-transferases (GST) [33]. However, no studies have explored the effects of dietary choline on ROS clearing capacity and whether it can affect flesh WHC in animal muscle. It has heen reported that choline can increase GPx activity in rat liver [34]. A previous study demonstrated that dietary choline decreased SOD, GPx, GR and GST activities in the head kidney and spleen of juvenile Jian carp [35]. These studies demonstrated that dietary choline may increase the activities of antioxidant enzymes to improve muscle WHC in fish. This possibility is worth investigating.

Antioxidant enzyme activities were partly dependent on antioxidant enzyme gene mRNA levels in mice liver [36], which are regulated by NF-E2-related factor 2 (Nrf2) in fish [37]. When exposed to oxidative stress, Nrf2 dissociates from Kelch-like ECH-associated protein 1 (Keap1), translocates to the nucleus and induces transcription of antioxidant enzyme genes in terrestrial animals [36]. A study reported that fish had two types of Keap1 (Keap1a and Keap1b) [37], and previous study demonstrated that dietary choline could modulate Nrf2 and Keap1a mRNA expression in the head kidney and spleen of juvenile Jian carp [35]. Furthermore, a study demonstrated that the target of rapamycin (TOR) could regulate Nrf2 expression in rat liver [38]. In mammals, mTOR has emerged as a critical nutritional and cellular energy checkpoint sensor and regulator of protein synthesis by enhancing the activities of positive regulators of translation factors [39]. Moreover, protein kinase casein kinase 2 (CK2) emerged as a ubiquitous cellular signaling molecule that regulates TOR in human glioblastoma cells [40]. However, little information has been produced on the effects of choline on the Nrf2 pathway in fish muscle. In rats, choline could significantly increase serum insulin content [41], which improved CK2 expression in adipocytes [42] and activated TOR signaling in rainbow trout [43]. These studies indicated that choline may regulate fish muscle antioxidant enzyme activities through modulating their gene expression, which may relate to the CK2-TOR-Nrf2 signaling pathway. This possibility warrants further investigation.

Grass carp (*Ctenopharyngodon idella*) is an important economic freshwater species that is widely cultured over the world [44]. The production of cultured grass carp in China is estimated to be 4.78 million tons, and is ranked second among domestic cultured freshwater fish production in 2012 [45]. The dietary choline requirement of fingerling grass carp has been evaluated in fingerling grass carp [46], but the choline requirements of fish may vary with growth stage. In rainbow trout, based on weight gain, a requirement for choline of 0.12 g initial weight (4000 mg/kg diet) [47] was higher than that at 3.2 g initial weight (714 mg/kg diet) [48]. In addition, the requirements of nutrients may vary with the different indicators. For example, the dietary myo-inositol (MI) requirement of juvenile Jian carp based on muscle protein carbonyl content was estimated to be 853.8 mg MI/kg diet [49], which was higher than that based on percent weight gain (518.0 mg MI/kg diet) [50]. Therefore, it is necessary to study choline requirements of grass carp.

Our current research aimed to evaluate the influence of choline on fish growth and for the first time to explore the influence of choline on fish flesh quality. Furthermore, we measured the antioxidant enzyme gene mRNA levels, as well as the signaling molecules TOR, CK2, Keap1a, Keap1b and Nrf2 gene mRNA levels of fish muscle. The results from this experiment may provide partial explanation for choline-enhanced fish growth and flesh quality. The suitable choline requirements for young grass carp growth and flesh quality were also evaluated, which may be used in formulating commercial feeds for the intensive culture of grass carp.

## Materials and Methods

### 2.1. Experimental diets and design

The formulation of the basal diet is present in Table 1. In order to achieve the maximum growth of grass carp, the isonitrogenous basal diet was contains 30% crude protein [51]. Basal diet mixed with various amounts of choline chloride to provide graded levels of 0 (un-supplemented), 400, 750, 1100, 1450 and 1800 mg choline/kg diet. The final choline concentration were 197, 385, 770, 1082, 1436 and 1795 mg choline/kg diet analyzed by the method of Venugopal [52]. After being prepared completely, the diets were stored at −20°C as described by Wu et al. [10].

**Table 1:** Formulation and proximate composition of basal diet.

Ingredients	g/kg	Nutrients content	g/kg
Fish meal	37.50	Crude protein ^4^	295.10
Casein	248.10	Crude lipid ^4^	45.40
Gelatin	75.00	n-3 ^5^	10.00
DL-Met (99%)	1.40	n-6 ^5^	10.00
α-starch	240.00	Available phosphorus ^5^	5.90
Corn starch	236.20		
Fish oil	25.00		
Soybean oil	18.90		
Cellulose	50.0		
Ca(H_2_PO_4_)_2_	22.40		
Choline-free vitamin premix ^1^	10.00		
Mineral premix ^2^	20.00		
Choline chloride premix ^3^	15.00		
Ethoxyquin (30%)	0.50		

^1^ Vitamin premix (g kg^-1^ premix): retinyl acetate (500 000 IU g^-1^), 2.40; cholecalciferol (500 000 IU g^-1^), 0.40; D,L-a-tocopherol acetate (50%), 12.54; menadione (23%) 0.79; thiamine nitrate (98%), 0.04; calcium-D-pantothenate (98%), 2.43; pyridoxine hydrochloride (98%), 0.59; cyanocobalamin (1%), 0.81; folic acid (9.6%), 0.40; niacin (99%), 2.17; D-biotin (2%), 4.91; mesoinositol (99%), 19.19; riboflavin (80%), 0.55; ascorhyl acetate (93%), 7.16. All ingredients were diluted with corn starch to 1 kg.

^2^ Mineral premix (g kg^-1^ premix): MgSO_4_·H_2_O, 56.200; FeSO_4_·H_2_O, 22.900; CuSO_4_·5H_2_O, 0.020; ZnSO_4_·H_2_O, 0.630; MnSO_4_·H_2_O, 1.650; KI, 0.070; NaSeO_3_, 0.004. All ingredients were diluted with corn starch to 1 kg.

^3^ Choline chloride premix (mg kg^-1^ premix): choline chloride was added to obtain graded levels of choline. The final choline concentrations in each experimental diet were determined to be197, 385, 770, 1082, 1436 and 1795 mg choline/kg diet, respectively.

^4^ Crude protein, crude lipid and total phosphorus contents were measured value.

^5^ Available phosphorus, n-3 and n-6 contents were calculated according to NRC (2011).

### 2.2. Feeding management

The procedures used in this study were approved by the University of Sichuan Agricultural Animal Care Advisory Committee. Before the experimental period, fish were acclimated themselves to the experimental system for 4 weeks [53]. Then, fish with an average initial body weight of 266.9 ± 0.6 g were stocked in 18 experimental cages (1.4*1.4*1.4m) with 30 fish per cage. In accordance with Tang et al. [54], each diet was randomly assigned to triplicate cages, and fish were fed 4 times daily for eight weeks, and uneaten feed was collected. During the experimental period, the treatment groups were under natural light cycle, dissolved oxygen was not less than 6.0 mg/L and pH was maintained at 7.0 ± 0.5 according to Tang et al. [54]. The water temperature was 26 ± 2°C.

### 2.3. Sample collection and analysis

At the end of experiment, after being starved for 12 h, fish were weighed and anaesthetized in a benzocaine bath (50 mg/L) as described by Deng et al. [55]. After sacrifice, they were manually filleted, and the muscle samples were obtained from the left side, frozen in liquid N_2_, and then stored at -80°C until analysis as described by Salmerón et al. [56]. Meanwhile, muscle samples were obtained from the right side of the same fish for analysis of flesh quality parameters as reported by Wu et al. [19]. The muscle cooking loss was measured as described by Brinker and Reiter [5]. The hydroxyproline concentration was assayed by hydrolysis in hydrochloric acid as described in Wu et al. [30]. The method of Zhou et al. [57] used to measure muscle moisture content (oven drying to a constant weight) and the protein (N-Kjeldahl×6.25) and lipid contents (solvent extraction with petroleum) were analyzed as described by Geurden et al. [58]. Muscle amino acid composition was analyzed using high-performance liquid chromatography (HPLC) as reported by Gan et al. [59]. Lactate content was measured using enzymatic colorimetric analysis according to Hultmann et al. [60]. Additionally, the fluorimetric method of Li et al. [61] was used to measure the activity of cathepsins.

The malondialdehyde (MDA) concentration was measured using the method of Alirezaei et al. [62] with the thiobarbituric acid reaction. Protein carbonyl (PC) concentration was evaluated by the method of Armenteros et al. [63] by the formation of protein hydrazones using 2, 4-dinitrophenylhydrazine. The anti-hydroxyl radical (AHR) capacity was measured using the Griess reagent, and the anti-hydroxyl radical (a-HR) capacity was analyzed using the Fenton reaction as described in Jiang et al. [64]. The anti-superoxide anion (ASA) capacity was determined using the Superoxide Anion Free Radical Detection Kit. Superoxide radicals were generated by the action of xanthine and xanthine oxidase; with an electron acceptor added, a coloration reaction is created using the gross reagent. The coloration degree is directly proportional to the quantity of superoxide anion in the reaction, such as in the methods described by Jiang et al. [64]. The GSH content was determined by measuring the formation of 5-thio-2-nitrobenzoate (TNB) according to Tang et al. [54]. The activity of Cu/Zn-SOD was assayed by measuring the decrease in the rate of cytochrome c reduction in a xanthine-xanthine oxidase superoxide generating system [59]. The GPx and GST activities were determined by measuring the rate of NADPH oxidation and monitoring the formation of an adduct between GSH and 1-chloro-2, 4-dinitrobenzene, respectively [49]. Catalase activity was determined by the decomposition of hydrogen peroxide according to Wu et al. [30]. GR activity was assayed as previously described by Wu et al. [30]. Total thiol-containing compound (T-SH) content was determined by the formation of 5-thio-2-nitrobenzoate followed by spectrophotometry at 412 nm. In addition, the muscle ROS content was measured using 2,7-dichlorodihydrofluorescein diacetate, which was oxidized to fluorescent dichlorofluorescein (DCF) as described in Rhee et al. [65].

### 2.4. Real-time quantitative PCR analysis

Total RNA was isolated from grass carp muscle using an RNAiso Plus Kit and electrophoresed on a 1% denaturing agarose gel to test the integrity [59]. Then, RNA was treated with DNase I to remove DNA contaminant and purified RNA reverse transcribed to cDNA. Specific primers for Cu/Zn-SOD, CAT, GPx, GR, GST, GCL, Nrf2, Keap1a, Keap1b, TOR and CK2 genes, as well as appropriate annealing temperatures are presented in Table 2. Due to results from our previous experiments (data not shown), we choose the β-actin (the most stable of the reference genes analyzed) as the reference gene. The amplification efficiencies of the reference gene and target genes were approximately 100% [59]. The expression results were analyzed using the 2^-ΔΔCT^ method after verification as described by Salmerón, et al. [56].

**Table 2:** Real-time quantitative PCR primers.

Gene	Sequences of primers	Annealing temperature (°C)	Accession number
Cu/Zn-SOD	F:CGCACTTCAACCCTTACA R:ACTTTCCTCATTGCCTCC	61.5	GU901214
CAT	F:AAGTTCTACACCGATGAGG R:CCAGAAATCCCAAACCAT	58.7	FJ560431
GPx	F:GGGCTGGTTATTCTGGGC R:AGGCGATGTCATTCCTGTTC	61.5	EU828796
GST	F:TCTCAAGGAACCCGTCTG R:CCAAGTATCCGTCCCACA	58.4	EU107283
GR	F:GTGTCCAACTTCTCCTGTG R:ACTCTGGGGTCCAAAACG	59.4	JX854448
GCL	F:CACGCTGCCAGAATACAA R:ATCACCACCTTTTCGCC	56.9	KF998103
Nrf2	F:CTGGACGAGGAGACTGGA R:ATCTGTGGTAGGTGGAAC	62.5	KF733814
Keap1a	F:TTCCACGCCCTCCTCAA R:TGTACCCTCCCGCTATG	63.0	KF811013
Keap1b	F:TCTGCTGTATGCGGTGGGC R:CTCCTCCATTCATCTTTCTCG	57.9	KJ729125
TOR	F:TCCCACTTTCCACCAACT R:ACACCTCCACCTTCTCCA	61.4	JX854449
CK2	F:CCCCAACCACAGTGACCT R:TCCCTGCTGATACTTCTCC	57.9	KF914143
β-Actin	F:GGCTGTGCTGTCCCTGTA R:GGGCATAACCCTCGTAGAT	61.4	M25013

### 2.5. Calculations and statistical analysis

Statistical analyses were carried out using SPSS 18.0 (SPSS Inc., Chicago, IL, USA); all data are presented as the means ± SD and were analyzed by ANOVA [59]. Significant differences were considered at the *P* < 0.05 level, and Tukey’s test was used to identify differences among experimental groups. We calculated the dietary choline requirement as described by Feng et al. [66].

## Results

### 3.1. Growth performance, muscle protein, lipid and moisture content

**Fig 1:**
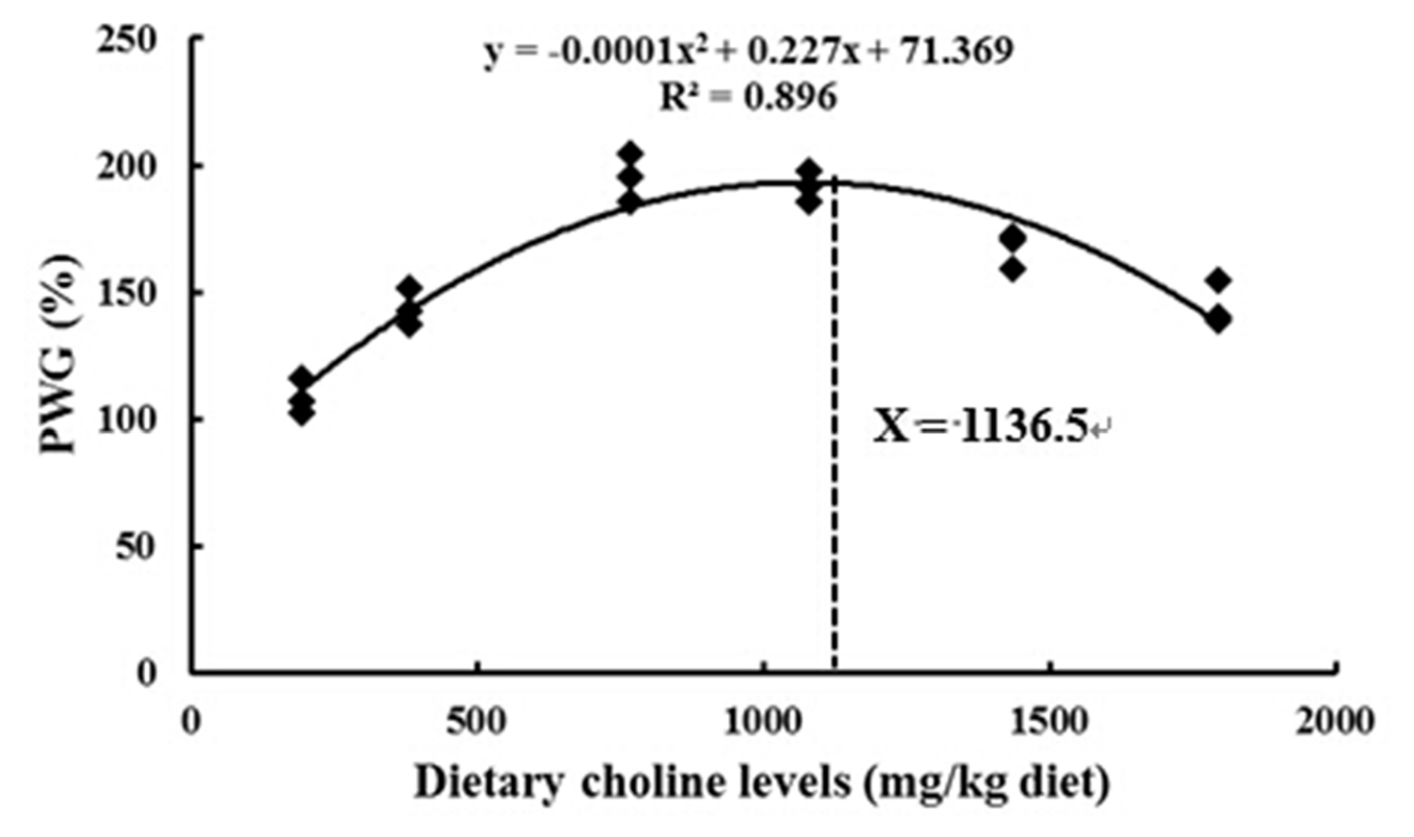
Relationship between PWG (percentage weight gain) and dietary choline levels, where Xopt represents the optimum dietary choline level for the maximum PWG of grass carp. Results are shown as means ± SD (n = 3).

As shown in Table 3, fish fed diets with 770 mg/kg and 1082 mg/kg choline had the highest final body weight (FBW) and percentage weight gain (PWG) (*P* < 0.05), followed by fish fed diets with 1436 mg/kg choline, and was lowest in fish fed diets with 197 mg/kg choline. The feed efficiency (FE) significantly increased when diets were supplementated with 770–1082 mg/kg choline (Table 3). Feed intake (FI) significantly increased with the increase of choline from 197 to 770 mg/kg diet and declined with a further supplementation (*P* < 0.05) (Table 3). The optimum dietary choline requirement level was estimated at 1136.5 mg/kg by a quadratic regression on the basis of PWG (Fig 1). As shown in Table 3, muscle composition was markedly affected by choline levels. Fish fed choline-supplement diets has a higher content of protein and lipid but a lower content of moisture. The content of protein was observed to be the highest in fish fed the diet with 770 mg/kg choline, followed by fish fed diets with 1082 mg/kg and 1436 mg/kg choline, and was lowest in fish fed diets with 197 mg/kg choline. The lipid content of fish muscle was the highest in the 770 mg/kg diet and 1082 mg/kg diet, whereas the moisture content of fish muscle was lowest in the 770 mg/kg diet and the 1082 mg/kg diet.

**Table 3:** Growth parameters and muscle proximate composition (%).

Dietary choline levels (mg/kg diet)						
	197	385	770	1082	1436	1795
IBW^1^	266.6±0.14^a^	267.1±0.33^a^	266.8±0.165^a^	267.1±0.38^a^	266.7±0.40^a^	267.2±0.07 ^a^
FBW^1^	554.7±19.0^a^	650.1±19.9^b^	787.1±24.8^d^	777.9±15.7^d^	711.4±17.6^c^	653.2±21.6^b^
PWG^1^	115.79±7.03^a^	151.35±7.3^b^	204.28±9.4^d^	197.2±6.02^d^	171.5±7.08^c^	154.6±8.78^b^
FI^1^	460.7±1.89^a^	603.9±0.95^c^	702.3±0.76^f^	680.9±1.59^e^	618.9±0.71^d^	554.6±0.50^b^
FE^1^	62.55±3.92^a^	63.42±3.36^a^	74.08±3.61^b^	75.01±2.17^b^	71.85±2.91^ab^	69.61±4.09^ab^
Moisture^2^	81.97±0.73^d^	79.08±0.97^bc^	76.60±0.70^a^	76.80±0.76^a^	78.02±0.27^b^	79.25±0.29^c^
Protein^2^	14.53±0.62^a^	16.41±0.73^b^	18.53±0.50^e^	17.78±0.51^de^	17.31±0.26^cd^	16.61±0.24^bc^
Lipid^2^	1.47±0.10^a^	1.81±0.13^b^	2.59±0.13^d^	2.72±0.18^d^	2.20±0.17^c^	1.69±0.13^ab^
Regression						
Y_PWG_ = – 0.0001x^2^ + 0.227x + 71.369				R^2^ = 0.896		*P* < 0.01
Y_FI_ = – 0.0003x^2^ + 0.598x + 385.020				R^2^ = 0.886		*P* < 0.01
Y_FE_ = – 1.402E-05x^2^ +0.033x + 55.437				R^2^ = 0.677		*P* < 0.01
Y_moisture_ = 6.174E-06x^2^–0.013x + 83.826				R^2^ = 0.823		*P* < 0.01
Y_protein_ = – 4.061E-06x^2^ + 0.009x + 13.311				R^2^ = 0.761		*P* < 0.01
Y_lipid_ = – 1.728E-06x^2^ + 0.004x + 0.795				R^2^ = 0.886		*P* < 0.01

^1^ Mean values of triplicate groups, with 30 fish in each group, and different superscripts in the same row are significantly different (*P* < 0.05).

^2^ Results are shown as means ± SD (n = 6), and the same row with different superscripts are significantly different (*P* < 0.05).

IBW: initial body weight (g/fish); FBW: final body weight (g/fish); PWG: percentage weight gain (%); SGR: specific growth rate (%/day); FI: feed intake (g/fish); FE: feed efficiency (%).

Weight gain (WG) = FBW–IBW

PWG = 100 × WG /IBW

FE = 100 × weight gain /feed intake.

### 3.2. Amino acid composition of muscle

As present in Table 4, dietary choline significantly affected amino acid composition in fish muscle. Threonine, cysteine, methionine, leucine and lysine contents were highest in the groups fed the 770 mg/kg diet and the 1082 mg/kg (*P* < 0.05) diet. Glutamic acid content was decreased by supplementation with 385–1083 mg/kg dietary choline (P < 0.05). Alanine content was highest in the group fed 1795 mg/kg choline and lowest in the group fed 385 mg/kg dietary choline (*P* < 0.05) However, dietary choline did not impact muscle aspartic acid, serine, glycine, valine, tyrosine arginine, phenylalanine or histidine contents (*P* > 0.05).

**Table 4:** Muscle amino acid content (g/100g dry) of fish fed the experimental diets for 8 weeks.

Dietary choline levels (mg/kg diet)						
	197	385	770	1082	1436	1795
Asp	8.86±0.11^a^	8.79±0.10^a^	8.90±0.04^a^	8.67±0.04^a^	8.70±0.16^a^	8.87±0.03^a^
Thr	3.45±0.02^a^	3.68±0.11^b^	3.92±0.01^c^	3.86±0.01^bc^	3.70±0.04^b^	3.40±0.03^a^
Ser	3.49±0.06^a^	3.40±0.04^a^	3.45±0.06^a^	3.49±0.08^a^	3.58±0.14^a^	3.52±0.09^a^
Glu	15.39±0.25^b^	14.46±0.36^ab^	13.38±0.04^a^	13.78±0.46^a^	14.49±0.25^ab^	15.35±0.15^b^
Gly	3.90±0.08^a^	3.88±0.10^a^	4.02±0.11^a^	4.00±0.12^a^	3.99±0.08^a^	3.92±0.05^a^
Ala	5.84±0.10^ab^	5.54±0.08^a^	5.58±0.10^ab^	5.58±0.03^ab^	5.57±0.08^ab^	5.87±0.01^b^
Val	4.10±0.03^a^	3.96±0.10^a^	3.99±0.04^a^	4.08±0.11^a^	4.06±0.14^a^	4.08±0.03^a^
Cys	0.47±0.00^a^	0.50±0.01^ab^	0.61±0.03^de^	0.64±0.01^e^	0.56±0.01^cd^	0.55±0.01^bc^
Met	2.57±0.05^a^	2.73±0.01^ab^	2.85±0.06^b^	2.82±0.06^b^	2.78±0.09^b^	2.64±0.01^ab^
Ile	3.92±0.06^c^	3.74±0.02^bc^	3.11±0.03^a^	3.46±0.13^b^	3.89±0.15^c^	3.94±0.01^c^
Leu	5.58±0.10^a^	5.89±0.19^ab^	6.40±0.17^b^	6.44±0.10^b^	6.06±0.07^ab^	5.73±0.15^a^
Tyr	3.21±0.05^a^	3.27±0.12^a^	3.24±0.11^a^	3.27±0.14^a^	3.34±0.13^a^	3.42±0.10^a^
Phe	3.22±0.13^a^	3.49±0.04^a^	3.52±0.04^a^	3.50±0.14^a^	3.59±0.06^a^	3.22±0.12^a^
Lys	6.43±0.10^a^	7.33±0.27^bc^	7.71±0.17^bc^	7.73±0.26^c^	7.26±0.28^abc^	6.84±0.20^ab^
His	1.53±0.04^a^	1.54±0.02^a^	1.54±0.07^a^	1.55±0.07^a^	1.50±0.01^a^	1.57±0.04^a^
Arg	5.06±0.07^a^	5.12±0.01^a^	5.30±0.08^a^	5.27±0.04^a^	5.13±0.05^a^	5.10±0.10^a^

Results are shown as means ± SD (n = 6), and a row with unlike superscript letters were significantly different (*P* < 0.05).

### 3.3. Flesh quality parameters

As presented in Table 5, the muscle cooking loss was higher in fish fed the choline deficient and excess diets, and the lowest in fish supplemented with 770 and 1082 mg/kg choline (*P* < 0.05). In contrast to cooking loss, muscle shear force was the highest in groups fed 770–1436 mg choline/kg diet (*P* < 0.05) (Table 5). The hydroxyproline content was also improved by 770–1082 mg/kg choline supplementation (*P* < 0.05) (Table 5). Cathepsin B and cathepsin L activities were the lowest with 770–1082 mg/kg choline supplementation (*P* < 0.05). With choline levels from 190 mg/kg to 770 mg/kg, the pH value decreased (*P* < 0.05), and the lowest pH value was found in the 770 mg/kg and 1082 mg/kg groups (*P* < 0.05) (Table 5). The lactate content significantly increased with choline levels from 197 mg/kg to 770 mg/kg (*P* < 0.05) (Table 5).

**Table 5:** Flesh quality parameters of grass carp fed the experimental diets for 8 weeks.

Dietary choline levels (mg/kg diet)						
	197	385	770	1082	1436	1795
Cooking loss	15.87±0.53^c^	15.08±0.61^bc^	11.81±0.97^a^	11.33±0.94^a^	14.08±0.96^b^	15.79±0.60^c^
Shear force	1.50±0.06^a^	1.60±0.05^b^	1.71±0.05^c^	1.73±0.03^c^	1.61±0.04^c^	1.55±0.04^ab^
Hydroxyproline	0.66±0.00^a^	0.71±0.01^bc^	0.75±0.01^c^	0.75±0.01^c^	0.72±0.01^bc^	0.69±0.02^ab^
pH	6.18±0.01^cd^	6.15±0.03^bc^	6.07±0.01^a^	6.06±0.02^a^	6.14±0.02^b^	6.19±0.03^d^
Cathepsin B	5.65±0.19^c^	5.01±0.23^b^	4.55±0.23^a^	4.57±0.15^a^	4.83±0.14^ab^	5.38±0.24^c^
Cathepsin L	2.27±0.13^c^	1.80±0.12^b^	1.38±0.08^a^	1.46±0.09^a^	1.75±0.08^b^	2.17±0.06^c^
lactate	1.45±0.08^a^	1.87±0.11^b^	3.02±0.12^c^	3.06±0.14^c^	1.93±0.07^b^	1.45±0.07^a^
Regression						
Y_cooking loss_ = 6.734E-06x^2^–0.013x + 18.563				R^2^ = 0.775		*P* < 0.01
Y_shear force_ = – 3.075E-07x^2^ + 0.001x + 1.400				R^2^ = 0.722		*P* < 0.01
Y_pH_ = 1.966E-07x^2^–0.0004x + 6.257				R^2^ = 0.820		*P* < 0.01
Y_cathepsin B_ = 1.538E-06x^2^–0.003x + 6.115				R^2^ = 0.802		*P* < 0.01
Y_cathepsin L_ = 1.262E-06x^2^–0.002x + 2.644				R^2^ = 0.892		*P* < 0.01
Y_Lactate_ = – 2.438E-06x^2^ + 0.005x + 0.576				R^2^ = 0.849		*P* < 0.01

Results are shown as means ± SD (n = 6), and a row with unlike superscript letters were significantly different (*P* < 0.05).

### 3.4. Muscle antioxidant parameters

**Fig 2:**
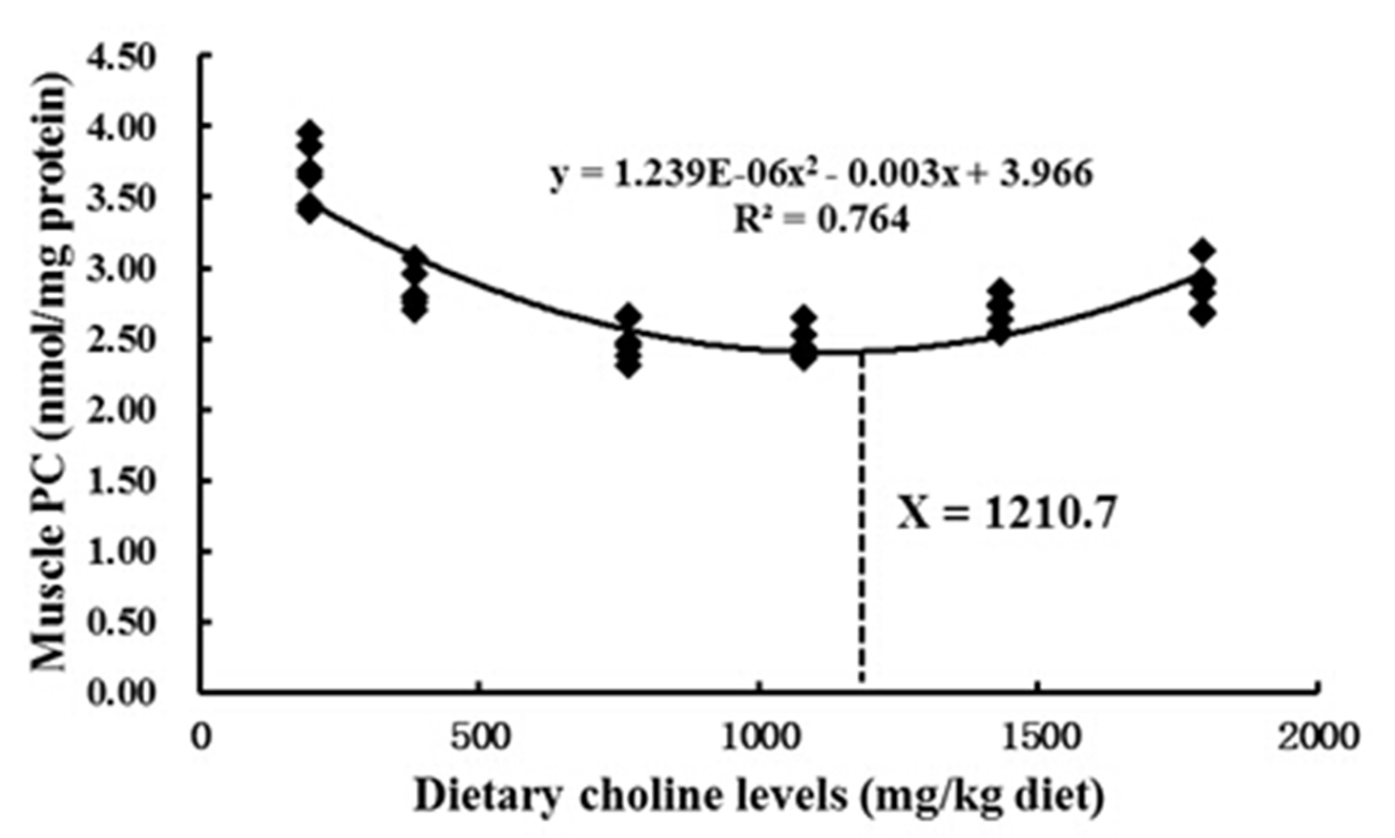
Relationship between muscle PC (protein carbonyl) content and dietary choline levels, where Xopt represents the optimum dietary choline level for the minimum PC content of grass carp. Results are shown as means ± SD (n = 6).

The ROS, MDA, PC and GSH contents, as well as the activities of ASA, AHR, Cu/Zn-SOD, CAT, GPx, GST and GR, in fish muscle are presented in Table 6. The ROS content decreased with 197–1082 mg/kg choline supplementation, and the lowest ROS was found in the 770 mg/kg and 1082 mg/kg groups (*P* < 0.05). MDA and PC contents notably declined with choline supplementation from 197 mg/kg to 1082 mg/kg, and the lowest content was found in the 770 mg/kg and 1082 mg/kg groups (*P* < 0.05). However, fish fed diets with 770 mg/kg and 1082 mg/kg choline achieved the highest ASA and AHR capacities. The activity of Cu/Zn-SOD was the highest when choline level was 770 mg/kg or 1082 mg/kg (*P* < 0.05). The GPx activity had a similar pattern to Cu/Zn-SOD. The GST activity and GSH content were enhanced incrementally with choline supplementation from 197 mg/kg to 1082 mg/kg and then declined with further supplementation (*P* < 0.05). However, the activities of CAT and GR were not influenced by dietary choline (*P* > 0.05). As presented in Fig 2, quadratic regression analysis on muscle PC content estimated the dietary requirement for choline to be 1210.7 mg/kg diet for grass carp (266.5–787.1 g) under current experimental conditions.

**Table 6:** ROS (% treatment 1), MDA (nmol/mg protein), PC (nmol/mg protein) contents; ASA (U/g protein), AHR (U/mg protein), Cu/Zn-SOD (U/mg protein), CAT (U/mg protein), GPx (U/mg protein), GST (U/mg protein), GR (U/g protein) activities and GSH (mg/g protein) content^1^.

Dietary choline levels (mg/kg diet)						
	197	385	770	1082	1436	1795
ROS	100.00±7.72^d^	64.58±4.75^b^	53.79±4.88^a^	48.93±4.78^a^	66.25±4.12^b^	81.20±7.30^c^
MDA	4.18±0.26^d^	3.74±0.30^bc^	2.79±0.18^a^	2.67±0.16^a^	3.59±0.25^b^	4.02±0.25^cd^
PC	3.66±0.22^c^	2.84±0.14^b^	2.48±0.14^a^	2.45±0.11^a^	2.70±0.10^ab^	2.85±0.16^b^
ASA	65.94±5.69^a^	89.18±8.18^bc^	111.79±8.69^d^	119.25±6.81^d^	91.89±7.30^c^	78.84±6.03^b^
AHR	242.27±11.20^a^	265.70±10.29^a^	304.53±9.53^b^	299.23±27.00^b^	263.05±9.85^a^	246.38±11.16^a^
Cu/Zn-SOD	6.91±0.50^a^	7.97±0.57^bc^	9.35±0.65^e^	8.96±0.53^de^	8.11±0.54^cd^	7.13±0.49^ab^
CAT	1.51±0.11^a^	1.47±0.11^a^	1.47±0.10^a^	1.50±0.11^a^	1.47±0.07^a^	1.45±0.11^a^
GPx	81.91±3.59^a^	107.37±4.35^b^	135.77±4.77^c^	139.80±8.75^c^	111.41±5.24^b^	88.12±4.89^a^
GST	77.90±6.83^a^	92.36±6.33^bc^	98.50±7.08^cd^	108.29±5.51^d^	91.06±6.75^bc^	81.22±5.68^ab^
GR	20.10±1.65^a^	19.78±2.07^a^	21.91±1.31^a^	20.87±1.59^a^	20.80±1.80^a^	21.28±1.38^a^
GSH	7.20±0.46^a^	8.62±0.61^bc^	10.49±0.82^d^	11.52±0.81^d^	8.77±0.35^c^	7.59±0.64^ab^
Regression						
Y_ROS_ = 6.126E-05x^2^–0.126x + 114.680				R^2^ = 0.794		*P* < 0.01
Y_MDA_ = 2.127E-06x^2^–0.004x + 4.952				R^2^ = 0.778		*P* < 0.01
Y_PC_ = 1.239E-06x^2^–0.003x + 3.966				R^2^ = 0.764		*P* < 0.01
Y_ASA_ = - 6.707E-05x^2^ + 0.137x + 44.342				R^2^ = 0.804		*P* < 0.01
Y_AHR_ = - 8.768E-05x^2^ + 0.171x + 214.629				R^2^ = 0.667		*P* < 0.01
Y_Cu/Zn-SOD_ = - 3.339E-06x^2^ + 0.007x + 5.887				R^2^ = 0.702		*P* < 0.01
Y_GPx_ = - 8.293E-05x^2^ + 0.165x + 54.970				R^2^ = 0.904		*P* < 0.01
Y_GST_ = - 3.738E-05x^2^ + 0.074x + 66.451				R^2^ = 0.661		*P* < 0.01
Y_GSH_ = - 5.608E-06x^2^ + 0.011x + 5.237				R^2^ = 0.772		*P* < 0.01

^1^ ROS: reactive oxygen species, MDA: malondialdehyde, PC: protein carbonyl, ASA: anti-superoxide anion, AHR: anti-hydroxyl radical, Cu/Zn-SOD: copper/zinc superoxide dismutase, CAT: catalase, GPx: glutathione peroxidase, GST: glutathione-S-transferase, GR: glutathione reductase, GSH: glutathione. Results are shown as means ± SD (n = 6), and a row with unlike superscript letters were significantly different (*P* < 0.05).

### 3.5. Gene expression in fish muscle

**Fig 3:**
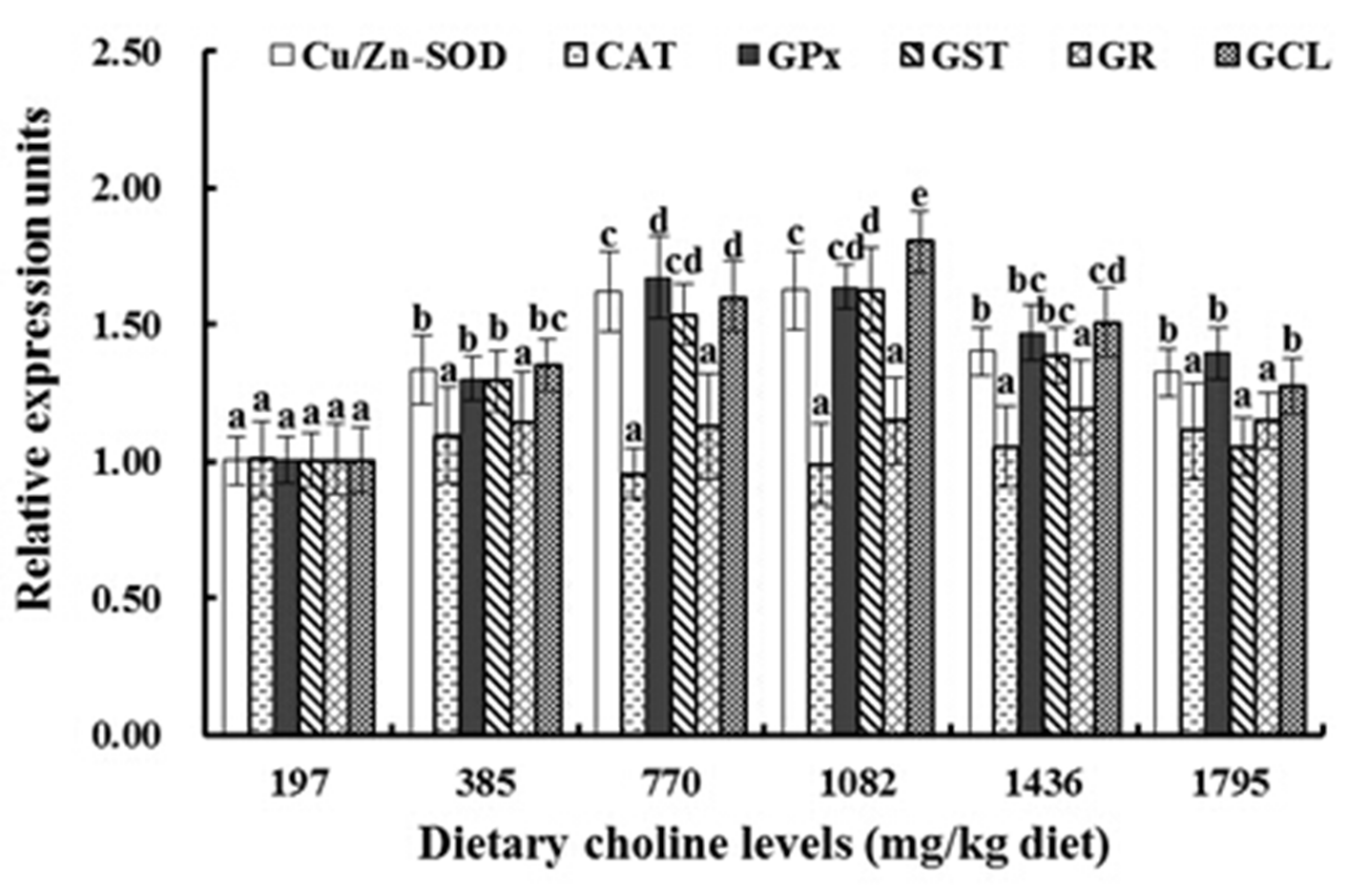
Relative gene expression levels of copper/zinc superoxide dismutase (Cu/Zn-SOD), catalase (CAT), glutathione peroxidase (GPx), glutathione S-transferase (GST), glutathione reductase (GR) and glutamate-cysteine ligase (GCL) in muscle of fish fed the experimental diets for 8 weeks. Results are shown as means ± SD (n = 6). Bars lacking a common superscript differ significantly (*P* < 0.05).

**Fig 4:**
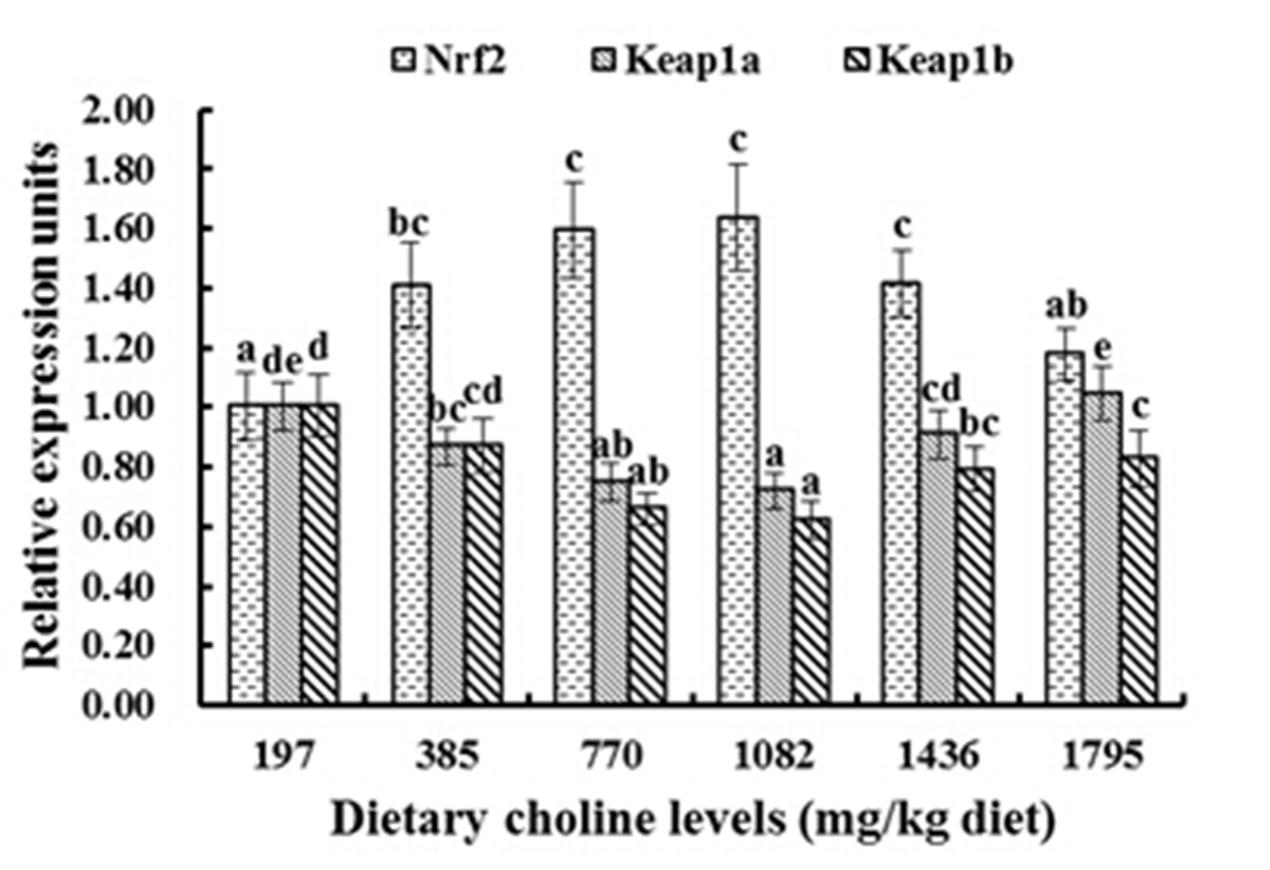
Relative gene expression levels of NF-E2-related factor 2 (Nrf2), Kelch-like- ECH-associated protein 1a (Keap1a) and Kelch-like- ECH-associated protein 1b (Keap1b) in muscle of fish fed the experimental diets for 8 weeks. Results are shown as means ± SD (n = 6). Bars lacking a common superscript differ significantly (*P* < 0.05).

**Fig 5:**
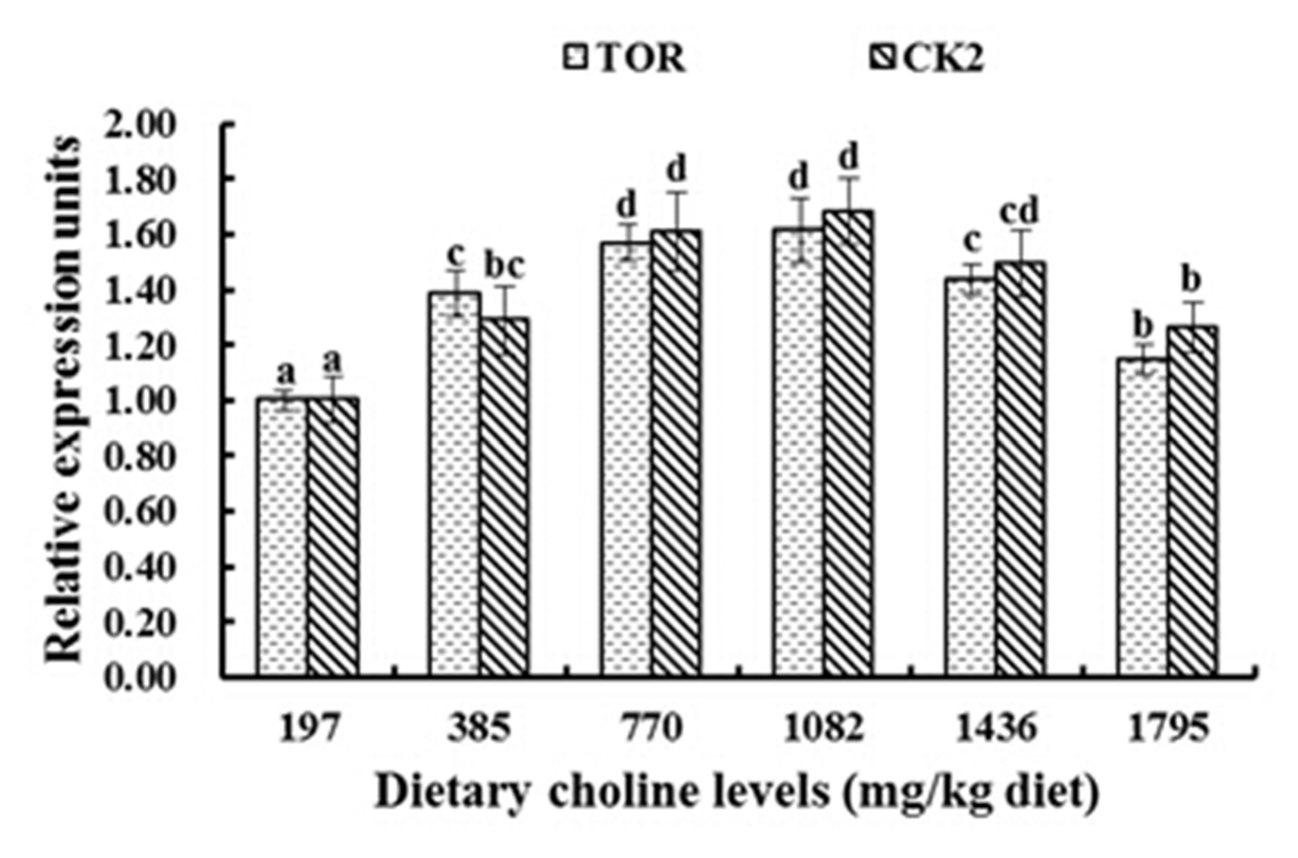
Relative gene expression levels of target of rapamycin (TOR) and casein kinase 2 (CK2) in muscle of fish fed the experimental diets for 8 weeks. Results are shown as means ± SD (n = 6). Bars lacking a common superscript differ significantly (P < 0.05).

As presented in Fig 3, with increasing choline up to 770 mg/kg, relative expression of Cu/Zn-SOD, GPx and GST genes in the muscle significantly increased, and reached a maximum in the 770 mg/kg and 1082 mg/kg groups (*P* < 0.05), but then declined with dietary choline greater than 1082 mg/kg (*P* < 0.05). With diets supplemented with 1082 mg/kg choline, fish had the highest GCL mRNA levels, followed by the 770 mg/kg and 1436 mg/kg groups, and then followed by the other diets (*P* < 0.05) (Fig 3). However, the relative expression of CAT and GR were not significantly different among these treatment groups (*P* > 0.05) (Fig 3). With increasing choline supplementation, relative expression of Nrf2 significantly increased, and reached a maximum in 770 mg/kg and 1082 mg choline/kg diet groups (*P* < 0.05). Relative expression of Keap1a and Keap1b had an opposite pattern compared to Nrf2 (*P* < 0.05) (Fig 4). With 197–770 mg/kg choline supplementation, relative expression of TOR significantly increased and reached a maximum in the 770 mg/kg and 1082 mg/kg groups, and then decreased with dietary choline levels higher than 1082 mg/kg (*P* < 0.05) (Fig 5). The CK2 mRNA expression was similar to TOR, and was the highest with 770 mg/kg and 1082 mg/kg choline supplementation (*P* < 0.05) (Fig 5).

## Discussion

### 4.1. Choline improved growth performance and muscle nutrient composition of fish

In our current study, grass carp fed diets with 385–1795 mg/kg choline had significantly higher growth performance, which suggested that suitable dietary choline could improve fish growth. On one hand, enhancement of growth performance in fish may be a consequence of feed intake. Our result showed that the optimum dietary choline enhanced FI, and correlation analysis showed the PWG of young grass carp was positively correlated with FI (r_FI_ = +0.974, P < 0.01), indicating that choline stimulates feed intake thereby improving fish growth. On the other hand, another study showed that fish growth is an accurate and important tool in studying fish feed efficiency [67]. Meanwhile, when feed intake increases, a lower percentage of the feed intake is used for fueling basal metabolism and this improves feed efficiency in fish [58]. The present study has demonstrated that optimal dietary choline significantly improved FE, and correlation analysis showed that FE was positively correlated with FI (r = +0.809, P = 0.051), suggesting that choline-elevated FE may occur through stimulating FI in fish. In addition, the growth of fish is partly attributed to muscle nutrient deposition [66]. The main chemical components of fish muscle are water, protein, and lipids, which make up approximately 98% of the total mass of the flesh [68]. Additionally, the lipid content in muscle has been recognized as a determinant of flavor, juiciness and texture for terrestrial animals as well as fish, which impacts on consumer perceptions [13]. Furthermore, increase in feed intake stimulates lipid deposition in fish tissues, muscle included [58]. Our current study also demonstrated that optimal dietary choline could improve muscle fat content of young grass carp, which was higher in the groups fed the 770 mg/kg diet and the 1082 mg/kg diet. As stated above, our data together corroborate the growth-promoting effects of choline caused primarily by higher feed intake. Moreover, choline-supplemented diets could have significantly improved protein accretion of juvenile shrimp *Penaeus monodon* [69]. The present study has demonstrated that optimal dietary choline significantly improved muscle protein content, as well as increased the muscle threonine, methionine, cysteine, leucine and lysine contents (Table 4). In addition to the nutrient composition, the firmness, pH value and WHC are the important flesh quality characters [70]. Thus, we next assayed the effects of dietary choline on firmness, pH value and WHC of young grass carp muscle.

### 4.2. Choline improved flesh quality of fish

#### 4.2.1. Choline improved flesh firmness of fish

Flesh firmness is an important flesh quality parameter. A decline in flesh firmness makes fish meat unappealing to consumers [70]. Shear force is a reliable biomarker that represents the flesh firmness of fish [71]. In the current study, shear force of muscle markedly increased with dietary choline from 197 to 1082 mg/kg and decreased thereafter in young grass carp (Table 5). Another study indicated that flesh firmness was positively correlated with the collagen content of muscle in Atlantic salmon [19]. In addition, the muscle collagen content could be quantified by the hydroxyproline concentration [30]. Meanwhile, Engel and Bächinger [72] reported that hydroxyproline had a positive effect on collagen stability. As shown in Table 5, supplementation with 385–1436 mg/kg choline markedly improved hydroxyproline content in fish muscle. There is a positive correlation between muscle shear force and hydroxyproline concentration (r = +0.974, *P* < 0.01), indicating that choline-enhanced flesh firmness may in part due to increasing collagen concentration. Additionally, the enhanced flesh firmness in this study may be because choline decreased cathepsin activity in fish muscle. Cathepsins (such as cathepsin B and L, two of important proteolytic enzymes) are one of the enzymatic system components that are involved in fish muscle degradation [73]. Elevated cathepsin activity results to faster muscle degradation in Atlantic salmon [22]. Fish muscle degradation could reduce the firmness of the fillet in rainbow trout [20]. In the current study, optimum dietary choline supplementation decreased cathepsin B and L activities (Table 5). Correlation analysis indicated that muscle shear force was negatively related to cathepsin B (r = -0.964, *P* < 0.01) and L (r = -0.972, *P* < 0.01) activities, suggesting that increment of flesh firmness may partly attribute to optimum dietary choline decreased cathepsin B and L activities. In conclusion, optimal dietary choline could increase fish flesh firmness possibly through increasing collagen content and inhibition of cathepsin B and L activities. In addition, the development of muscle firmness is likely to be compartmentalized by muscle pH in fish [74]. Thus, we next investigated the pH of the fish muscle.

#### 4.2.2. Choline decreased flesh pH value of fish

Post-mortem muscle pH is another important flesh quality parameter in fish [75]. High pH value makes the fish flesh more sensitive to spoilage and decreases shelf-life [26]. It was reported that deterioration of Atlantic cod flesh quality is partly due to proteolysis of muscle protein, and proteolytic activities were significantly lower at a pH of 6.0 [76]. Our study showed that the grass carp post-mortem muscle pH was higher in the choline-deficient group (6.18) and choline excess group (6.19) and significantly decreased with dietary choline levels from 385 mg/kg to 1082 mg/kg (6.06) (Table 5), which indicated that appropriate dietary choline decreased the fish muscle pH to prevent flesh quality deterioration in fish. The decreased muscle pH by optimal choline may have contributed to the increased anaerobic metabolism-induced lactate production in fish muscle. Another study has demonstrated that lactate accumulation in Atlantic salmon postmortem muscle resulted from anaerobic metabolism, which induced a pH decline [77]. In our current study, the lactate content was increased with appropriate dietary choline supplementation in fish muscle (Table 5). Additionally, there was a negative correlation among the pH and lactate concentration (r = –0.995, *P* < 0.01), which suggested that optimal dietary choline might decrease pH value partly through increasing lactate content in fish muscle. Apart from firmness and pH value, water-holding capacity (WHC) is another key flesh quality characteristic that affects consumers’ perception [28]. It was reported that the flesh WHC was correlated to antioxidant status in fish muscle [30]. Due to this possibility, we next explored how muscle WHC and antioxidant status varied with choline levels.

#### 4.2.3. Choline improves fish flesh WHC and is partly attributable to elevated muscle antioxidant status

Water-holding capacity (WHC) is a key flesh quality, which could be evaluate by cooking loss [78]. Decreased cooking loss was observed from elevating the WHC of cod (*Gadus morhua*) muscle [78]. In this study, we firstly demonstrated that optimal dietary choline significantly decreased muscle cooking loss, which indicated that optimal choline could improve flesh WHC (Table 5). Another study showed that the improvement in muscle WHC could be attributed to protection of muscle structural integrity, which resulted from deceasing oxidative damage in chicks [79]. In fish, PC and MDA contents are widely used as indices for protein and lipid oxidation damage, respectively [80]. However, there is no information about how dietary choline affects fish muscle oxidation damage. In our current research, optimal choline supplementation significantly reduced the MDA and PC content of young grass carp muscle (Table 6). There is a positive correlation among muscle cooking loss and their MDA and PC contents (r_MDA_ = +0.993, *P* < 0.01; r_PC_ = +0.768, *P* = 0.075), indicating that optimal choline improved WHC, which may be partly due to reducing muscle oxidation damage in fish muscle. Oxidation damage was caused by ROS, and OH˙ and O^2-^ are two toxic ROS in fish [33]. In this study, optimal dietary choline significantly decreased muscle ROS content, and increased ASA and AHR activities (Table 6). There was a negative correlation among muscle ROS content and ASA and AHR activities (r_ASA_ = –0.961, *P* < 0.01; r_AHR_ = –0.900, *P* < 0.05). Our data indicated that appropriate choline could increase O^2—^cleaning capacity and OH˙-cleaning capacity in fish muscle. GSH is an effective non-enzymatic antioxidant that neutralizes ROS [33]. In the present study, optimal choline significantly elevated GSH content (Table 6) and decreased cooking loss (Table 5) in the fish muscle. There was a negative correlation between muscle cooking loss and GSH content (r_GSH_ = -0.980, *P* < 0.01), indicating that optimal dietary choline significantly improved flesh WHC perhaps because of enhanced GSH content in fish muscle. However, there has been no study conducted to research how choline impacts on fish muscle GSH content. Another study in fish demonstrated that muscle GSH content was positively correlated with GR activity [81]. However, in our present study, GR activity was not affected by dietary choline (Table 6). In addition, the increased GSH content was attributed to the synthesis of new glutathione molecules [82]. It has been reported that glutamate-cysteine ligase (GCL) is a key component of the enzyme that is involved in GSH synthesis [71]. In our study, optimal choline supplementation increased the mRNA levels of GCL (Fig 3). Moreover, muscle GSH content was significantly correlated with GCL expression (r_GCL_ = +0.944, *P* < 0.05). The data implied that choline increased GSH content partly by increasing the GCL gene expression in fish muscle. In addition, ROS can be eliminated by antioxidant enzymes (such as SOD, GPx and GST) in fish [33]. However, there is no more information about how dietary choline affects antioxidant enzyme activity in fish muscle. In our current study, optimal dietary choline significantly increased Cu/Zn-SOD, GPx and GST activities (Table 6). The improvement of antioxidant enzyme activities by choline was perhaps because choline can elevate muscle methionine content. In a study in rats, methionine proved to improve kidney SOD, GPx and GST activities [83]. In rat liver, choline re-methylated homocysteine to methionine [84]. In the present study, optimal dietary choline significantly improved muscle methionine content (Table 4). However, dietary choline failed to influence the activity of CAT in fish muscle (Table 6). The insignificant change in the CAT activity may be attributed to the increase in other antioxidant enzymes, such as GPx [85], because H_2_O_2_ is eliminated by CAT and GPx also participates in the reduction of H_2_O_2_ [82]. There was a negative correlation among muscle cooking loss and antioxidant enzyme activities (r_Cu/Zn-SOD_ = –0.950, *P* < 370 0.01; r_GPx_ = –0.974, *P* < 0.01; r_GST_ = –0.929, *P* < 0.01) in young grass carp. Therefore, we conjectured that choline-enhanced muscle WHC may be due to choline up-regulating Cu/Zn-SOD, GPx and GST activities. Therefore, our current study demonstrated that the enhancement of flesh WHC by choline was partly attributed to the improvement in antioxidant status. In addition, the improvement in antioxidant status was positively correlated to the expression of antioxidant enzyme genes [55].

### 4.3. Choline regulated antioxidant enzyme gene expression: a link to Nrf2 signaling pathways in fish muscle

In the current study, optimal choline supplementation significantly elevated the mRNA levels of Cu/Zn-SOD, GPx and GST in fish muscle (Fig 3). Correlation analysis showed that Cu/Zn-SOD, GPx and GST activities were positively related to their respective mRNA levels (r_Cu/Zn-SOD_ = +0.918, *P* = 0.01; r_GPx_ = +0.879, *P* < 0.05; r_GST_ = +0.972, *P* < 0.01). These data suggested that suitable choline elevated antioxidant enzyme activities partly due to up-regulating their gene expression in fish muscle.

Nrf2 has been demonstrated to be a critical transcription factor that regulates antioxidant enzyme gene expression [37]. Chen et al. [36] reported that the up-regulation of Nrf2 expression increased SOD and GPx mRNA expression in mouse liver. In the present study, optimal dietary choline significantly up-regulated muscle Nrf2 mRNA levels (Fig 4). Correlation analysis showed that the expression of Cu/Zn-SOD, GPx and GST were positively correlated with the gene expression level of Nrf2 (r_Cu/Zn-SOD_ = +0.953, P < 0.01; r_GPx_ = +0.904, P < 0.05; r_GST_ = +0.978, P < 0.01), implying that choline up-regulated the expression level of Cu/Zn-SOD, GPx and GST partly by increasing the Nrf2 gene expression in fish muscle. In addition, another study demonstrated that the promotion of Nrf2 nuclear translocation could elevate the expression of antioxidant genes in mice liver [86]. Keap1 was identified as an Nrf2-binding protein, which depresses Nrf2 translocation to the nucleus [87]. Moreover, another study reported that fish had two types of Keap1, Keap1a and Keap1b [37]. In the current study, the mRNA levels of Keap1 a and Keap1b in fish muscle were down-regulated by optimal choline levels (Fig 4), suggesting that optimal choline may have increased the Nrf2 activity to up-regulate Cu/Zn-SOD, GPx and GST gene mRNA levels through down-regulating both Keap1a and Keap1b mRNA level in fish muscle.

Moreover, Nrf2 expression could be activated by TOR [88]. A previous study demonstrated that elevated TOR expression could up-regulate Nrf2 expression in endothelial cells [89]. In our present study, optimal choline up-regulated TOR mRNA levels in fish muscle (Fig 5). There was a positive correlation between the expression of Nrf2 and the expression of TOR in young grass carp muscle (r = +0.997, *P* < 0.01), suggesting that choline up-regulating Nrf2 mRNA levels may be partly through elevating the mRNA levels of TOR in fish muscle. Moreover, choline-enhanced TOR mRNA levels may be partly due to the up-regulation of CK2. In vitro, the up-regulation of CK2 caused the up-regulation of the expression of TOR in human glioblastoma cells [40]. The data presented here showed that optimal dietary choline significantly improved CK2 mRNA levels in fish muscle (Fig 5). Correlation analysis showed that the relative gene expression of TOR was significantly correlated with CK2 mRNA expression (r = +0.961, *P* < 0.01), showing that choline up-regulating TOR mRNA expression may be partly through up-regulating CK2 mRNA expression in fish muscle. For the above-mentioned results, it is apparent that optimal choline up-regulated TOR expression to enhance Nrf2 gene expression through increasing the expression of CK2 mRNA in fish muscle. However, the exact mechanisms through which choline regulates Nrf2-related signaling molecules remains largely unknown and needs additional investigation.

### 4.4. Differential effect of dietary choline on antioxidant status among muscle and immune organs in fish

In our current study, optimal dietary choline significantly increased ASA, AHR, Cu/Zn-SOD, GPx and GST activities and glutathione content in fish muscle (Table 6). However, our previous study in juvenile Jian carp found that dietary choline could decrease antioxidant enzyme activities and glutathione content in the spleen and head kidney [35]. Those observations indicated that the regulatory effects of choline on muscle antioxidant status were different from that in immune organs. The reasons for the discrepancies remain unknown but might be attributable to three reasons. First, lipid content of the spleen (4.05±0.14) and head kidney (3.67±0.17) are higher than muscle (2.59±0.13) in young grass carp, which indicated that fish spleen and head kidney is more susceptible to oxidative damage than muscle. Second, a study in rabbits demonstrated that the kidney was one of the most important organs involved in choline metabolism and was the organ primarily affected in choline deficiency relative to muscle [90]. Third, choline deficiency could induce production of ROS in rat kidney [31]. A study demonstrated that ROS was adaptive to up-regulation of antioxidant enzyme activities [91]. Therefore, as important functioning organs, head kidney and spleen are crucial organs for the survival of the fish and may be able to compensate to improve antioxidant enzyme activities when dietary choline is deficient.

### 4.5. Choline requirements of young grass carp

The above data clearly demonstrated that optimum dietary choline could improve fish growth and flesh quality. The dietary choline requirements of young grass carp (266.5–787.1 g) based on PWG and muscle PC content were established to be 1136.5 mg choline/kg and 1210.7 mg choline/kg diet, respectively. The results indicated that choline requirement of fish for antioxidant was higher than that for growth. Similar results of other nutrients like myo-inositol has been reported in juvenile Jian carp [49].

## Conclusions

In summary, the present study showed that optimal dietary choline elevated flesh firmness, which may be related to collagen content and cathepsin activities. Moreover, optimal dietary choline reduced pH value partly by increasing lactate concentration in fish muscle. Optimal dietary choline improved fish flesh WHC partly through enhancing muscle antioxidant status by a) increasing muscle GSH content, which might be partly due to up-regulation gene expression of GCL, and b) up-regulating Cu/Zn-SOD, GPx and GST activities, which might be partly ascribed to up-regulation of their gene expression. Moreover, the gene expression may be regulated by several signaling molecules (Nrf2, Keap1 a, Keap1b, CK2 and TOR) that are involved in the Nrf2 signaling pathway. Interestingly, the effect of choline on antioxidant status in fish muscle was different from our study in immune organs [35]. All of the results provide a partial mechanism for the positive effect of dietary choline on flesh quality. However, the exact mechanisms of choline’s effects require further investigation. In addition, the dietary choline requirements of young grass carp (266.5–787.1 g) based on PWG and muscle PC content were 1136.5 mg/kg and 1210.7 mg/kg diet, respectively.
